# Musashi1 as a potential therapeutic target and diagnostic marker for lung cancer

**DOI:** 10.18632/oncotarget.1034

**Published:** 2013-05-21

**Authors:** Xiao-Yang Wang, Huina Yu, R. Ilona Linnoila, Laodong Li, Dangyu Li, Biwen Mo, Hideyuki Okano, Luiz O. F. Penalva, Robert I. Glazer

**Affiliations:** ^1^ Cell and Cancer Biology Branch, Center for Cancer Research, National Cancer Institute, National Institutes of Health, Bethesda, MD, USA; ^2^ Division of Respiratory Diseases, Guilin Medical University Hospital, Guilin, Guangxi, P.R. China; ^3^ Division of Respiratory Diseases, Nan Xi Shan Hospital, Guilin Medical University Hospital, Guilin, Guangxi, P.R. China; ^4^ Department of Physiology, Keio University, Tokyo, Japan; ^5^ Children's Cancer Research Institute, Department of Cell and Structural Biology, University of Texas Health Sciences Center at San Antonio, San Antonio, TX, USA; ^6^ Department of Oncology, Georgetown University, and Lombardi Comprehensive Cancer Center, Washington, DC, USA

**Keywords:** Musashi1, lung cancer, shRNA, β-catenin, notch, numb

## Abstract

Lung cancer remains one of the leading causes of cancer-related deaths worldwide with a 5-year survival rate of less than 20%. One approach to improving survival is the identification of biomarkers to detect early stage disease. In this study, we investigated the potential of the stem cell and progenitor cell marker, Musashi1 (Msi1), as a diagnostic marker and potential therapeutic target for lung cancer. Functional studies in A549 bronchioalveolar carcinoma and NCI-H520 squamous cell carcinoma cells revealed that Msi1 was enriched in spheroid cultures of tumor cells and in the CD133+ cell population. Downregulation of Msi1 by lentivirus-mediated expression of an Msi1 shRNA reduced spheroid colony proliferation. Growth inhibition was associated with reduced nuclear localization of β-catenin and inhibition of the processing of intracellular Notch. In primary lung cancer, Msi1 protein expression was elevated in 86% of 202 tissue microarray specimens, and Msi1 mRNA was increased in 80% of 118 bronchoscopic biopsies, including metastatic disease, but was rarely detected in adjacent normal lung tissue and in non-malignant diseased tissue. Msi1 was expressed in a diffuse pattern in most tumor subtypes, except in squamous cell carcinomas, where it appeared in a focal pattern in 50% of specimens. Thus, Msi1 is a sensitive and specific diagnostic marker for all lung cancer subtypes.

## INTRODUCTION

Lung cancer is the most common cause of cancer-related mortality worldwide [1], and despite modern diagnostic and therapeutic advances, the 5-year survival rate following resection has improved only in patients diagnosed with early stage disease. At present, only one out of eight patients diagnosed with lung cancer can be effectively treated due to the prevalence of rapidly metastatic disease [2], and therefore, a key aspect of improving survival is the diagnosis of early disease. Lung cancer is often diagnosed by bronchoscopic biopsy, and bronchial brushing and bronchial lavage have been used as adjuncts to bronchoscopy for histological, cytological [3] and molecular [4] analysis. With the advent of genomics and personalized medicine, the diagnosis and treatment of lung cancer is expected to improve [5, 6].

Musashi1 (Msi1) is an RNA-binding protein that was initially identified in *Drosophila melanogaster* by its ability to regulate sensory organ development and asymmetric cell division [7, 8]. In mammalian cells, Msi1 regulates translation in stem and progenitor cell populations of several tissues [8-10]. Msi1 is a member of a family of more than 800 RNA-binding proteins that regulate a multitude of processes associated with the post-transcriptional regulation of gene expression, and when aberrantly expressed can lead to diseases such as cancer [11]. Among the documented targets of Msi1-mediated translational repression are Numb, a negative regulator of Notch [12], p21^Waf1^, a negative regulator of cyclin-dependent kinases [13], poly(A) binding protein [14], Doublecortin, a factor involved in neuronal migration [15], Robo3, a protein that controls neural guidance [16] and Apc, a negative regulator of Wnt signaling [17]. A genome-wide analysis of Msi1 targets revealed >60 additional mRNA targets, many of which were related to cancer progression [18]. In malignant tissues, Msi1 is highly expressed in gastric [19], gallbladder [20], colorectal [21], endometrial [22] and lung [23] cancer. In breast cancer [24], increased Msi1 expression correlated with metastatic disease and poor survival.

In addition to its usefulness as a cancer biomarker, Msi1 may serve as a potential therapeutic target. This has been suggested by lentivirus-mediated ‘knockdown’ (KD) of Msi1 in breast cancer [24, 25] and medulloblastoma cells [26], resulting in inhibition of proliferation and tumor xenograft growth.

In the present study, we report that Msi1 KD in A549 and H520 lung cancer cells inhibited the proliferation of the stem-like spheroid tumor cells that was associated with inhibition of Notch and Wnt signaling. Analyses of lung cancers indicated that Msi1 protein or RNA expression served as a highly sensitive diagnostic marker in >80% of >300 lung cancers irrespective of histological subtype.

## RESULTS

### Msi1 is enriched in spheroid cultures of lung cancer cells

**Figure 1:**
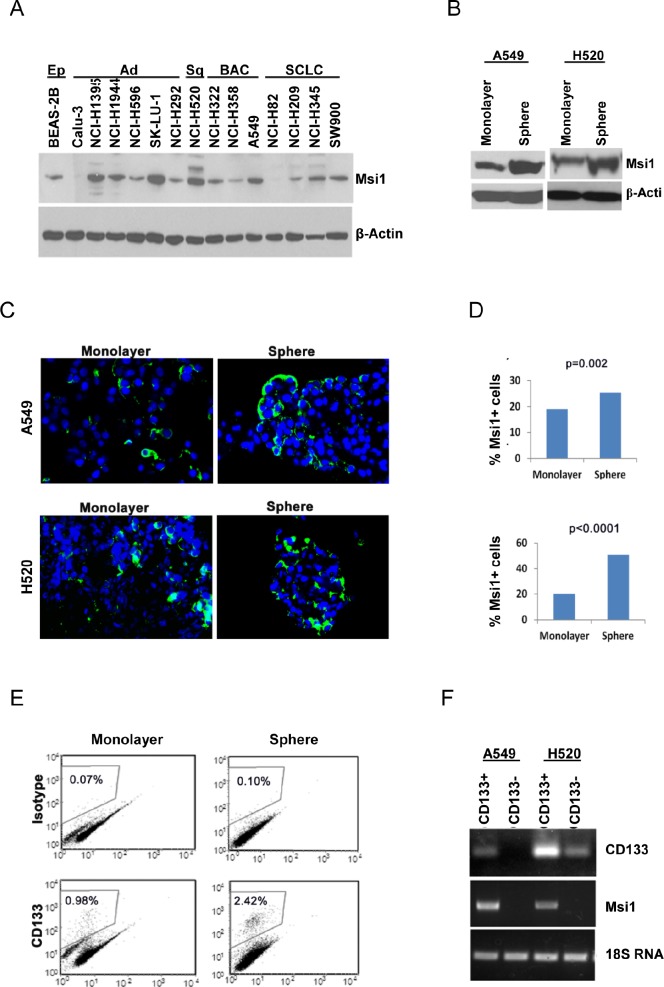
Msi1 expression is enriched in lung cancer stem/progenitor cells (A) Msi1 expression detected by western blotting is present in 12/14 lung cancer cell lines, as well as in immortalized lung epithelial cell line BEAS-2B. (B) Msi1 protein expression determined by western blotting is increased in spheroid cultures of A549 and H520 cells vs. monolayer cultures. (C, D) Msi1 expression determined by immunofluorescence is increased in spheroid cultures of A549 and H520 cells vs. monolayer cultures. Msi1 expression differed significantly between spheroid and monolayer cultures by the two-sided paired t test. Green, Msi1; blue, DAPI. (E) Spheroid cultures express a greater percentage of CD133+ cells vs. monolayer cultures. (F) CD133-enriched spheroid cultures exhibit greater expression of Msi1 mRNA vs. CD133-negative cells.

Msi1 expression was evaluated by western blotting in 14 lung cancer cell lines and one immortalized airway epithelial cell line (Figure 1A). Msi1 was expressed in 12/14 lung cancer cell lines, with high levels in about half of them, as well as in the epithelial cell line. To determine if Msi1 was enriched in stem-like cells, A549 and H520 cells were grown as spheroid cultures in defined medium in ultra-low attachment plates or under standard conditions as monolayers. Spheroid cultures contained about a two-fold enrichment of Msi1in comparison to monolayers (Figure 1B), and detection of Msi1 by immunofluorescence revealed a similar finding (Figure 1C,D). Since CD133 has been widely used as a lung cancer ‘stem cell’ marker [27-29], monolayer and spheroid cultures of A549 and H520 cells were sorted for CD133 and Msi1 mRNA levels determined. A representative CD133 profile for A549 cells indicated that CD133+ cells comprised 2.4% of the cells grown spheroids and 1% of the cell grown as monolayers (Figure 1E). Analysis of Msi1 RNA levels in CD133+ and CD133- A549 and H520 spheroid cultures indicated that CD133+ cells were enriched for Msi1 mRNA (Figure 1F).

### Lentivirus-mediated ‘knockdown’ of Msi1 inhibits spheroid colony proliferation

**Figure 2:**
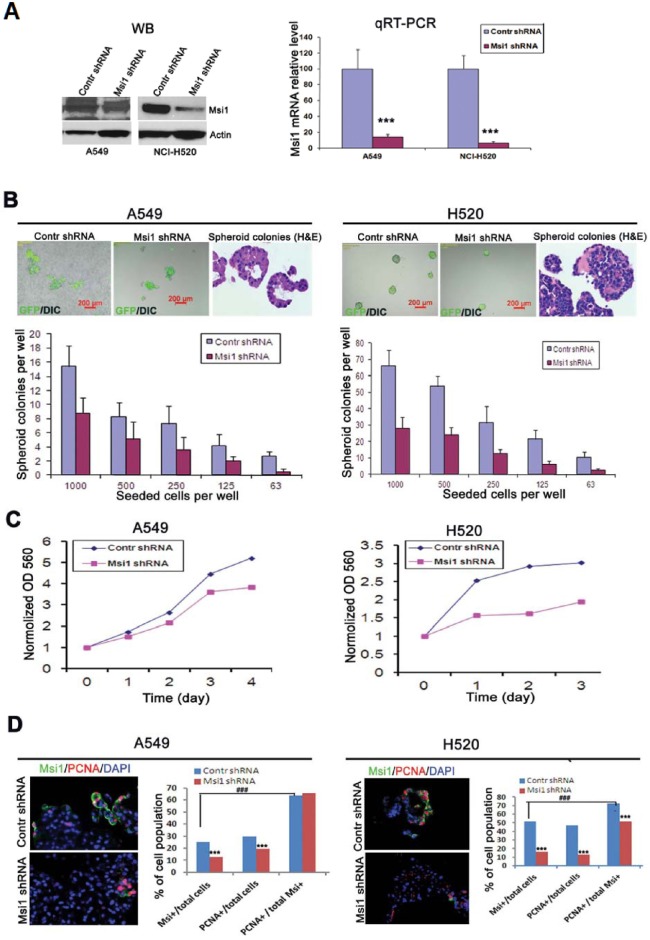
Reduction of Msi1 by RNA interference inhibits spheroid colony formation (A) Msi1 mRNA levels were “knocked-down” (KD) in A549 and H520 cells by transduction with a lentivirus-expressed shRNA. *Left panel*, Msi1 protein determined by western blotting. *Right panel*, Msi1 mRNA levels determined by qRT-PCR showing significant reduction of mRNA expression after transduction by a lentivirus-expressed shRNA vs. control shRNA (two-sided paired t test). (B) Quantitation of spheroid colony formation by detection of GFP fluorescence expressed by the transduced lentivirus. Colony formation was determined in serial dilutions of cells grown for 10 days in ultralow attachment plates in defined serum-free medium. Colony number was significantly decreased in A549 and H520 cells following Msi1 KD. The morphology of colonies of A549 cells resembled glandular epithelium (*H&E*), whereas, colonies of H520 cells exhibited a squamous cell carcinoma phenotype. (C) Msi1 KD significantly inhibited the growth of A549 and H520 cells. (D) Msi1 KD significantly reduced the percentage of Msi1^+^ and PCNA^+^ A549 and H520 cells vs. the percentage of PCNA^+^ cells in the total cell population; χ2 test; *** p<0.0001 vs. control (*Contr*) shRNA group; ### p<0.0001.

The role of Msi1 in the proliferation of A549 and H520 cells in spheroid culture was examined following lentivirus-mediated expression of an Msi1 shRNA [24]. RNA interference resulted in reduction of Msi1 protein and >90% reduction of Msi1 RNA in A549 and H520 cells in comparison to a control shRNA (Figure 2A), and 10 days post-transduction, 50% or more inhibition of colony formation was evident in both cell lines (Figure 2B). Interestingly, H520 cells, which resembled a squamous cell morphology, were inhibited to a greater extent than A549 cells, which resembled differentiated glandular cells (Figure 2B, H&E stained panels). Proliferation of dissociated spheroid cells expressing the Msi1 shRNA continued to grow at a reduced rate when grown in monolayer culture (Figure 2C). Determination of the co-expression of the proliferation marker PCNA and Msi1 following Msi1 KD revealed that Msi1 and PCNA expression were reduced by 50% and 31%, and by 69% and 72%, in A549 and H520 cells, respectively (Figure 2D).

### Msi1 KD reduces Notch and Wnt signaling

**Figure 3:**
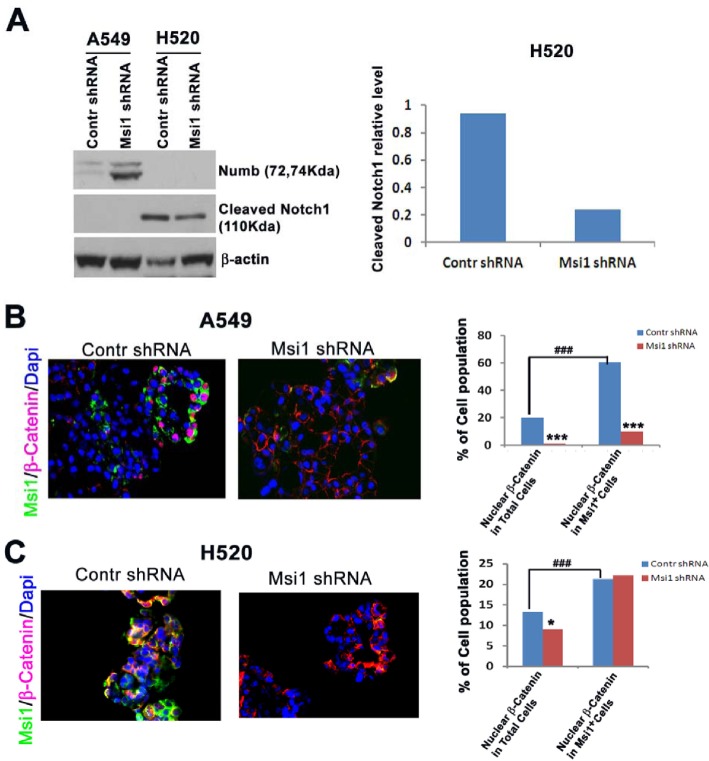
Msi1 KD impairs Notch processing and Wnt pathway activation (A) Msi1 KD in H520, but not A549 cells reduces intracellular Notch. Numb expression increased in A549 cells following Msi1 KD, but intracellular Notch was below the level of detection. (B) Msi1 KD reduces nuclear β-catenin translocation in A549 cells and (C) H520 cells. Immunofluorescent staining for Msi1 and β-catenin indicated significantly greater reduction of nuclear β-catenin in Msi1+ A549 cells vs. control shRNA-treated cells; χ2 test, * p<0.05, *** p<0.0001. The percentage of nuclear β-catenin was greater in Msi1+ cells vs. the total cell population; χ2 test, ### p<0.0001.

Msi1 was previously reported to inhibit Wnt pathway activation, as well as Notch activation through the negative regulator Numb [12, 30] in breast cancer and mammary epithelial cells [25]. Following Msi1 KD, there were significant differences in the regulation of these pathways between A549 and H520 cells. A549 and H520 cells responded differently to Msi1 KD with respect to Notch and Wnt signaling. Numb was markedly increased in A549 cells, but was undetectable in H520 cells, whereas, intracellular Notch1 was inhibited in H520 cells and was undetectable in A549 cells (Figure 3A). This suggests that Notch regulation may be more relevant to the growth of H520 cells than A549 cells. Since Numb is a cofactor for Notch ubiquitination [31], it is likely involved in the regulation of another yet to be identified target associated with proliferation. To assess the effects of Msi1 KD on canonical Wnt signaling, cells were analyzed for the nuclear translocation of β-catenin [32]. Msi1 KD inhibited β-catenin translocation by >85% in Msi1+ A549 cells (Figure 3B), whereas in H520 cells, localization was unaffected (Figure 3C), despite both cells lines having a relatively high percentage of nuclear β-catenin.

### Expression patterns of Msi1 in human lung cancers

**Figure 4:**
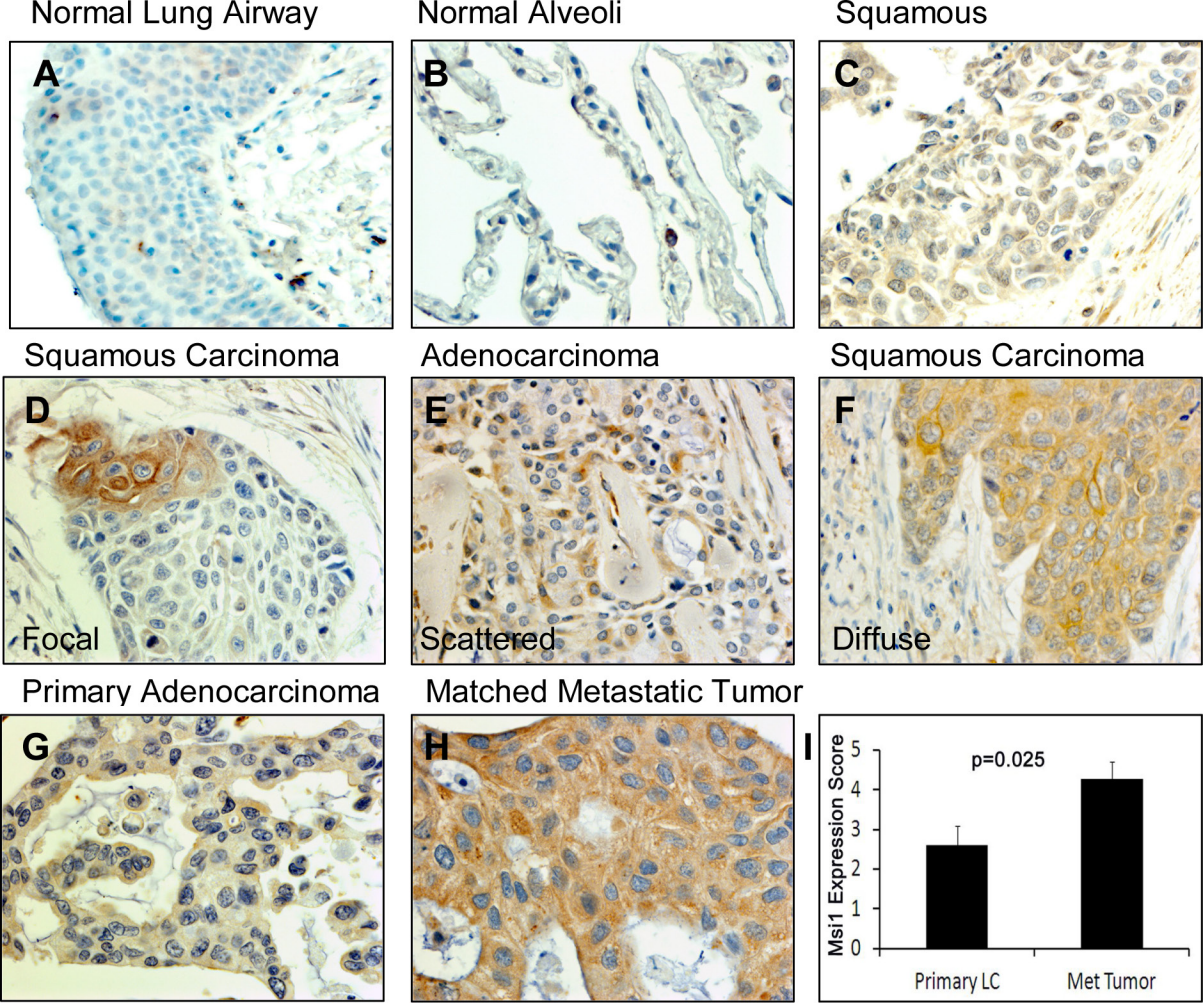
Msi1 expression patterns in lung cancers (A) Normal airway epithelium contained few Msi1^+^ cells (arrows). (B) Normal lung alveolus did not express Msi1. (C) Negative Msi1 expression in a squamous cell carcinoma. (D) Focal expression of Msi1 in a squamous cell carcinoma. (E) Scattered Msi1 expression in an adenocarcinoma. (F) Diffuse Msi1 expression in a squamous cell carcinoma. (G) Weak Msi1 expression in an adenocarcinoma and (H) high Msi1 expression in a paired metastatic lesion from (G). (I) Msi1 expression in paired primary and metastatic lung adenocarcinomas. Msi1 expression was significantly greater in metastatic lesions; two-sided paired t test, p=0.025.

To evaluate the usefulness of Msi1 as a lung cancer biomarker, Msi1 localization was examined in nonmalignant and lung cancer tissue microarrays by IHC (Figure 4, Table 1). Msi1 was weakly expressed in scattered cells in airway epithelium (Figure 4A) and was not present in the normal alveolus (Figure 4B). Lung tumors expressed Msi1 in a focal (Figure 4D), scattered (Figure 4E) or diffuse (Figure 4F) pattern in 86% of 202 lung cancer specimens irrespective of age, gender, histology and stage of disease (Table 1). With the exception of squamous cell carcinomas, ≥50% of tumors expressed Msi1 in a diffuse pattern (Figure 4F, Supplementary Table 1). Msi1 was often expressed at higher levels in lung tumor metastases than in the primary tumor, but did not reach statistical significance (*P*=0.159, N=15) (Figure 4G-I). Msi1 expression was also assessed by qRT-PCR in 102 lung cancers and 16 non-malignant lung tissues collected by bronchoscopic biopsy (Table 2). Similar to the tissue microarray findings (Table 1), 80% of lung cancers were Msi1+, irrespective of age, gender, histology, degree of differentiation or stage of disease, but was detectable in only 2 of 16 non-malignant specimens (Supplementary Table 2).

**Table 1:** Msi1 protein expression in lung cancer tissue microarray samples. Msi1 expression was analyzed by IHC. Ad, adenocarcinoma; BAC, bronchioloalveolar carcinoma; Ad-Sq, adenosquamous carcinoma; Sq, squamous cell carcinoma; LCLC, large cell lung carcinoma; SCLC, small cell lung carcinoma; Other, mucoepidermoid carcinoma or malignant mesothelioma. * χ2 test

Feature	No. Cases	% Msi1+	P -value
Age:			0.200*
<60	114	87	
≥60	98	86	
Gender:			0.111*
Male	160	88	
Female	42	79	
Histology:			0.381*
Ad	53	81	
BAC	20	95	
Ad-Sq	4	75	
Sq	81	91	
LCLC	15	80	
SCLC	25	80	
Other	4	75	
Total	202	86	
Stage:			0.240*
I~II	90	90	
III~IV	34	82	

**Table 2:** Msi1 mRNA expression in lung cancer bronchoscopic biopsy specimens. Msi1 mRNA was determined by qRT-PCR. Ad, adenocarcinoma; Sq, squamous cell carcinoma; SCLC, small cell lung carcinoma; Other, mucoepidermoid carcinoma or malignant. mesothelioma. * χ2 test

Feature	No. Cases	% Msi1+	P-value
Age:			0.769*
<60	54	82	
≥60	48	79	
Gender:			0.895*
Male	84	80	
Female	18	78	
Histology:			0.355*
Ad	17	77	
Sq	47	77	
SCLC	27	85	
Other	11	82	
Total	102	80	
Differentiation:			0.889*
Well-Moderate	24	75	
Poor	51	77	
Stage:			0.252*
I~II	11	64	
III~IV	78	83	

## DISCUSSION

In the present study, we evaluated the role of Msi1 in the proliferation of lung cancer cells and its utility as a diagnostic marker for lung cancers of varying histological subtypes. Msi1 expression was enriched in A549 and H520 lung cancer cells under cell culture conditions that promoted the formation of less differentiated spheroid colonies shown to be enriched for stem-like cells [33]. Similarly, cells sorted for CD133, a cell population enriched in tumor initiating cells [27-29], were further enriched for Msi1, in agreement with Msi1 as a stem cell or early progenitor cell marker , and consistent with its enrichment in CD133+ breast cancer “mammospheres” [24], and poorly differentiated oral squamous cell carcinomas [34]. Silencing Msi1 by RNA interference reduced spheroid colony proliferation, implicating Msi1 as a regulator of less differentiated stem-like cells. Thus, Msi1 expression in lung cancer cells has similar characteristics and function as previously noted for breast cancer [24], medulloblastoma [35], glioblastoma [36, 37] and colon cancer [38, 39] cells, and is consistent with the original observation of Msi1 being a marker for neural stem and progenitor cells [10, 40].

Msi1 is a positive regulator of Wnt and Notch signaling [25, 41] and several other pathways that remain to be explored [18]. A549, but not H520 cells exhibited a marked upregulation of Numb following Msi1 KD, consistent with the suppression of Numb translation by Msi1 [12] as previously observed in mammary epithelial and breast cancer cells [24, 25]. Although Numb was markedly increased by Msi1 KD in A549 cells, intracellular Notch was below detection in both control and Msi1 KD cells, making the association between Numb and Notch inconclusive. The increase in Numb in the presence of reduced Wnt signaling also suggests it is not a TCF/LEF target gene as previously noted [42]. Mechanistically, this could have resulted from a proliferin-mediated endocrine pathway as shown in mammary epithelial cells [25] or from blocking the translation of Apc, a negative regulator of canonical Wnt signaling [17]. Thus, Wnt pathway activation due to the nuclear translocation of β-catenin appears to play a dominant role in the proliferation of Msi1-expressing cells [24, 25, 41].

The majority of primary lung cancer biopsies exhibited varying patterns of Msi1 expression as previously noted in lung cancer specimens [23]. Lung cancer analyzed by tissue microarray or bronchoscopic biopsy expressed Msi1 protein or RNA in ≥80% of tumor specimens regardless of histological subtype. These findings are in general agreement with the report by Moreira et al. [23], although they found little or no Msi1 expression in large cell lung cancer and 40% Msi1+ adenocarcinomas. Other than patient variability and a smaller sample size, the reasons for disparity are not obvious. When Msi1 detection was combined with histopathological examination, the percentage of positive diagnoses was increased from 81% to 100% (results not shown), and the higher expression of Msi1 in metastatic disease may further increase its value as a diagnostic marker. The association between Msi1 expression in less differentiated lung cancer cells and proliferation also suggests that it may serve as a readily accessible marker of progression or response to therapy if it is present in sputum and bronchial washings from lung cancer patients.

Overall, our study shows that Msi1 regulates the proliferation of stem-like spheroid lung tumor cells in associated with Notch and Wnt signaling. Analyses of lung cancers indicated that Msi1 protein or RNA expression can serve as a highly sensitive diagnostic marker for lung cancer irrespective of the histological subtype.

## MATERIALS AND METHODS

### Cell Lines and Cell Culture

Human lung cancer cell lines Calu-3, NCI-H1395, NCI-H1944, NCI-H596, SK-LU-1, NCI-H292, NCI-H520, NCI-H322, NCI-H358, A549, NCI-H82, NCI-H209, NCI-H345, SW900, and immortalized human lung epithelial cell line BEAS-2B were obtained from the Tissue Culture Shared Resource, Lombardi Comprehensive Cancer Center, Georgetown University. NCI-H520 cells were cultured in RPMI1640 (Invitrogen, Grand Island, NY) supplemented with 10% fetal bovine serum (FBS) (Invitrogen). A549 cells were maintained in Dulbecco's- modified Eagle's medium (DMEM) (Invitrogen) supplemented with 10% FBS. All the cells were cultured at 37°C under 5% CO_2_ in a Forma Series II CO_2_ incubator (Thermo Scientific, Asheville, NC).

### Spheroid Cell Culture

Spheroid cell culture was carried out in ultra-low attachment plates (Costar, Corning, NY) as described [24]. Briefly, A549 and NCI-H520 cells growing as monolayer cultures were trypsinized with 0.05% trypsin-0.5 mM EDTA (Invitrogen), washed twice with PBS, counted and seeded into a 6-well plate at a density of 3,000 viable cells/ml or into a 96-well plate at serial dilutions of 1000 to 63 cells per 200 μl. Cells were grown in serum-free DMEM medium supplemented with 1X B27 (Invitrogen), 20 ng/ml epidermal growth factor (EGF) (Sigma) and 20 ng/ml basic fibroblast growth factor (FGF-2) (Invitrogen) at 37°C under 5% CO_2_. Spheroid clusters were counted and collected by gravity or gentle centrifugation (800 g, 10 sec) after 10 days, and dissociated in 0.05% trypsin-0.5 mM EDTA for 10-15 min by gentle pipetting. Cells were filtered through a 40-μm nylon mesh sieve (Falcon), analyzed microscopically for single cellularity and counted. Successive passages were plated at 1,000 cells/ml in 6-well plates. Generation 1 (G1) spheres, grown for 10 days, were fixed with 4% paraformaldehyde (PFA) for 2 hours, washed once. Spheres were collected, embedded in 4% agarose gel, and placed on ice for 10 minutes. The spheres were subsequently fixed with 4% PFA overnight prior to standard histological processing, sectioning, and hematoxylin and eosin (H&E) staining (American Histolabs Inc., Gaithersburg, MD).

### Lentivirus-mediated shRNA Expression

Msi1 expression was ‘knocked down’ (KD) using an shRNAmir GIPZ lentiviral vector targeting the sequence, 5'-CGT CCT GTA TCA TAT GTA AAT-3' in the 3'-UTR of Msi1 mRNA (Oligo ID #V2HS_280120; Open Biosystems, Huntsville, AL) [24]. TLA-HEK293T cells (Open Biosystems) were transfected with the Trans-Lentiviral Packaging Mix and pGIPZ transfer vector at 50% confluence using Arrest-In transfection reagent (Open Biosystems) according to the manufacturer's protocol. After incubation for 48-72 hr, the virus-containing supernatant was collected and centrifuged at 3,000 rpm for 20 min at 4°C, mixed 50:50 with fresh cell culture media, and used to transduce A549 and NCI-H520 cells. Lentivirus expressing a non-silencing control shRNA (shRNAmir, Open Biosystems) served as a negative control. Cells were selected for stable integration of the virus by incubation with 5 μg/ml puromycin (Sigma-Aldrich Corp. St. Louis, MO) for 10 days. The efficiency of integration was monitored by green fluorescent protein (GFP) co-expressed by the lentivirus.

### Growth Assay

Cells dissociated as a single cell suspension from first generation spheroid colonies were seeded in 96-well plates at 3,000 cells/well in 200 μl medium, and growth was determined after 24 to 96 hr by sulforhodamine B staining [25].

### Western Blotting

Western blotting was performed as described [25]. Primary antibodies included rat anti-Msi1 (1:1000) [45] (14H-1, Dr. Hideyuki Okano, Keio University, Tokyo, Japan), rabbit anti-Numb (1:1000, Cell Signaling Technology, Danvers, MA), rabbit anti-Cleaved Notch1 (Val1744) (D3B8) (1:1000, Cell Signaling Technology) and mouse anti-β-actin (1:3000, Sigma).

### Flow Cytometry

For CD133 detection, A549 and NCI-H520 cells were suspended at a concentration of 0.5-1.0×10^6^ cells/ml in ice-cold phosphate buffered saline (PBS) containing 3% fetal bovine serum (PBS/FBS). Cells were sorted with a Becton Dickinson FACSort system for CD133^+^ and CD133^−^ cell populations and analyzed with FCS Express V3 software (De Novo Software, Ontario, Canada) as described [24]. Cells were washed twice with PBS. CD133 and Msi1 mRNA expression were detected by RT-PCR in sorted cells [24].

### Dual Label Immunofluorescence

Spheroid colony slides were deparaffinized in xylene for 15 min, and rehydrated in graded ethanol solutions for 5 min each. Antigen retrieval was achieved by steaming slides for 10 min in 10 mM citrate buffer, pH 6.0. Dual label immunofluorescence was performed as previously described [25]. Primary antibodies were mouse monoclonal anti-human proliferating cell nuclear antigen (PCNA) (1:100, Dako North America, Inc. Carpentaria, CA), rabbit anti-beta-catenin (1:50, eBioscience, San Diego, CA), and anti-biotinylated Msi1 [45]. Antigens were retrieved as described above, and detected sequentially on sections by incubation for 1 hr with the first (Msi1) primary antibody followed by incubation with the appropriate FITC-conjugated streptavidin (eBioscience), followed by incubation for 1 hr with the second primary antibody followed by incubation with Alexa Fluor® 594-conjugated secondary antibody (Invitrogen). All incubations were at room temperature and sections were washed three times for 5 min each in PBS between each step. Sections were mounted in an anti-fading reagent with 4',6-diamidino-2-phenylindole (DAPI) (Invitrogen). Control slides were included in each analysis in which non-immune serum was substituted for primary antibodies and secondary antibodies individually.

### Tissue Microarrays

Lung cancer tissue microarray slides were obtained from Imgenex (San Diego, CA, Cat# IMH-358 and IMH-305), US Biomax (Rockville, MD, Cat# LC951) and Cybrdi (Rockville, MD, cat# CC04-01). There were a total of 202 cases of primary lung cancer, 16 metastatic carcinomas and 34 normal or non-tumor adjacent lung tissues. Patients included 160 males and 42 females between 26 and 81 years of age, with a median age of 59. The histology subtypes included 53 adenocarcinomas (Ad), 20 bronchioloalveolar carcinomas (BAC), 4 adenosquamous carcinomas (Ad-Sq), 81 squamous cell carcinomas (Sq), 15 large cell carcinomas (LCLC), 25 small cell lung carcinomas (SCLC) and 4 of other subtypes (3 mucoepidermoid carcinoma and 1 malignant mesothelioma). One hundred twenty four contained TNM stage data (Table S1).

### Immunohistochemistry (IHC)

Tissue microarray slides were baked at 62°C for 1 hr, deparaffinized in xylene for 15 min, and rehydrated in 100%, 95% and 70% ethanol for 5 min each. Antigen retrieval was achieved by steaming slides for 10 min in 10 mM citrate buffer, pH 6.0. Slides were washed three times in PBS and blocked for 1 hr in a buffer containing 10% goat serum in PBS, and incubated overnight at 4°C with a 1:1,000 dilution of rat anti-Msi1 antibody 14H-1 conjugated with biotin [45]. Slides were washed three times in PBS and antigen visualized with ABC Vectastain and DAB as substrate (Vector Labs, Burlingame, CA). Slides were counterstained with Harris-modified hematoxylin (Thermo-Fisher, Pittsburgh, PA) and mounted in Permount.

The Msi1 expression pattern criteria determined by IHC included: 1) ‘diffuse’ when almost all cells expressed the antigen, 2) ‘focal’ when isolated groups of Msi1^+^ cells were seen within a histological section and 3) ‘scattered staining’ when single cells were Msi1^+^. The level of Msi1 expression in lung cancer tissue was categorized by diffusion scores and intensity scores. The criteria of Msi1 diffusion expression scores were: 0, negative; 1, 1-30% positive cells, 2, 31-60% positive cells, and 3, >60% positive cells. Intensity scores were: 0, negative, 1, low intensity, 2, median intensity and 3, high intensity. Four high power fields (400X) were randomly chosen for counting Msi1^+^ cells in each core of the slide. A total of ~1000 cells per core per patient were analyzed. All slides were reviewed by one pathologist (Linnoila RI, M.D.) and one well-trained researcher in pathology (Wang XY, M.D., Ph.D.) blinded to the patients' clinical information.

### Clinical samples from bronchoscope biopsy

This study is a prospective analysis of 102 patients with histologically proven lung cancer and 16 non-cancer patients treated at Guilin Medical University Hospital and Affiliated Nan Xi Shan Hospital in China from March, 2011 to December, 2011. All cases were newly diagnosed and had not received anti-cancer therapy. The study was approved by the Guilin Medical University Review Board, and informed consent was obtained from all patients undergoing the procedures prescribed by the university ethics committee.

### Lung cancer patients

There were a total of 102 lung cancer patients comprising 84 males and 18 females ranging from 29 to 80 years of age (median = 59.5). Seventy-seven cases were non-small cell lung cancer (NSCLC) and 25 cases small cell lung cancer (SCLC); 60 patients were smokers. After a clinical examination, patients underwent CT scans for staging of the tumor. On the basis of clinical and radiological findings, 89 cases had staging data, and consisted of 27 cases of stage IV, 51 cases of stage III, 4 cases of stage II and 7 cases of stage I cancer (Table 2).

### Non-cancer control group

The control group consisting of 16 patients undergoing biopsy by fiberoptic bronchoscopy included 6 cases of tuberculosis, 6 cases with a diagnosis of bronchitis, 2 cases of pneumonia, 1 case of tuberculous pleurisy and 1 case of brochiectasis (Supplementary Table 2).

### Biopsy

All patients underwent bronchoscopy and at least 5 biopsy specimens were obtained. One to two specimens were snap frozen and stored at -80^0^ C for reverse transcription polymerase chain reaction (RT-PCR) analysis. The remaining specimens were immersed in buffered formalin for histopathological evaluation. RT-PCR was conducted after confirmation of the diagnosis.

### Semi-quantitative RT-PCR

Total RNA was isolated from the biopsy tissue using Trizol reagent (TakaRa Bio Inc., Dalian, China) according to the manufacturer's instructions. One microgram of template RNA was reverse transcribed to cDNA using PrimeScript II 1st Strand cDNA Synthesis Kit (TakaRa). PCR was carried out on 1 μl of the cDNA template, 1 μl (10 μM) forward/reverse primers using 2X GoldStar Taq MasterMix (CoWin Bioscience, Bejing, China) in final volume of 25 μl. The DNA thermal cycler conditions used were 94^0^C for 5 min (pre-denature), and 35 cycles of 94^0^C for 1 min, 56.2^0^C (for Msi1) or 62^0^C (for beta-actin) for 30s and 72^0^C for 45s, followed by a final extension of 72^0^C for 2 min. Six μl of each of Msi1 and β-actin PCR-amplified product was mixed and fractionated on a 2% agarose gel, which was then visualized by ethidium bromide staining using a JS-780 Gel Image Analysis System (Peiqing Sci Tech., Ltd, Shanghai, China). The primer pairs yield PCR products of 306 bp for Msi1 and 417 bp for β-actin, respectively. Primers for Msi1 were: 5'-GCT CGA CTC CAA AAC AAT TGA CC-3' (forward) and 5'-GGC TGA GCT TTC TTA CAT TCC AC-3' (reverse). Primer sequences for β-actin were: 5'-ACA GAG CCT CGC CTT TGC CGA TC-3'(forward) and 5'-TGG GTC ATC TTC TCG CGG TTG G-3'(reverse).

For quantitation of RT-PCR products, the integrated density of the PCR bands in the agarose gel was measured by a JS-780 Gel Image Analysis System. The relative level of Msi1 expression = Msi1 integrated density/β-actin integrated density. mRNA was obtained from cells in exponential growth and the PCR cycle numbers of Msi1 and β-actin during were based on the integrated density from 20 to 40 cycles.

### Statistical analysis

Data were analyzed with the Chi-Square test, Student's t test, Whitney-Mann U test and Kruskal-Wallis H test. Data were expressed as mean±standard deviation (SD) or percentage (%). A p-value of less than 0.05 was considered statistically significant. All statistical analyses were performed with the Statistical Package for Social Sciences (SPSS, version 11.0, Chicago, IL, USA).

## Supplementary Tables


