# Supplementary material for: Musashi1 as a potential therapeutic target and diagnostic marker for lung cancer
Source: Oncotarget. 2013 May 21;4(5):739–50. doi: 10.18632/oncotarget.1034 (PMC3742834; doi:10.18632/oncotarget.1034)
Supplement: Supplementary file 1 [file oncotarget-04-739-s001.pdf]

Supplementary Table 1. Msi1 expression patterns in lung cancer tissue microarray samples. Msi1 expression was determined by IHC. There was a significant association between Msi1 expression and malignancy, $P<0.001$, $\chi 2$ test

	No. Samples (%)				
	Negative	Focal	Scatter	Diffuse	Total
Ad	10 (19)	1 (2)	6 (11)	36 (68)	53 (100)
Ad-Sq	1 (25)	0 (0)	1 (25)	2 (50)	4 (100)
BAC	2 (10)	0 (0)	1 (5)	17 (85)	20 (100)
LCLC	1 (7)	0 (0)	3 (20)	11 (73)	15 (100)
SCLC	5 (20)	74 (16)	2 (8)	14 (56)	25 (100)
Sq	6 (7)	37 (46)	6 (7)	32 (39)	81 (100)
Other	1 (25)	0 (0)	0 (0)	3 (75)	4 (100)
Total	*26 (13)*	*42 (21)*	*19 (9)*	*115 (57)*	*202 (100)*

Supplementary Table 2. Msi1 expression in non-malignant lung bronchoscopic biopsy specimens. Msi1 RNA was determined by qRT-PCR. N/A, not available

Patient	Sex	Age	Smoker	Final Diagnosis	Histology	RT-PCR
1	M	63	1	Bronchitis	Chronic inflammation	0
2	M	61	N/A	Bronchitis	Necrosis	0
3	M	42	0	Bronchitis	Chronic inflammation	0
4	M	58	N/A	Bronchitis	Squamous cell metaplasia	0.982
5	M	71	N/A	Bronchitis	Chronic inflammation	0
6	M	73	N/A	Bronchitis	Chronic inflammation	0
7	F	59	0	Bronchiectasis	Chronic inflammation	0
8	M	54	0	Pneumonia	Chronic inflammation	0
9	M	53	1	Pneumonia	Chronic inflammation	0
10	F	58	0	Tuberculosis	Tuberculosis	0
11	M	52	1	Tuberculosis	Tuberculosis	0
12	M	26	N/A	Tuberculosis	Squamous cell metaplasia	0
13	F	71	0	Tuberculosis	Tuberculosis	0
14	F	38	0	Tuberculosis	Chronic inflammation	0
15	M	64	0	Tuberculosis	Chronic inflammation	0.025
16	M	79	1	Tuberculosis	Basal cell hyperplasia	0
